# Ironing out the Details: Untangling Dietary Iron and Genetic Background in Diabetes

**DOI:** 10.3390/nu10101437

**Published:** 2018-10-05

**Authors:** Mario A. Miranda, Heather A. Lawson

**Affiliations:** Department of Genetics, Washington University School of Medicine, Campus Box 8232, 660 South Euclid Ave, Saint Louis, MO 63110, USA; hlawson@genetics.wustl.edu

**Keywords:** iron, diabetes, diet, gene-environment interaction (GEI), insulin sensitivity, hepcidin, adipose

## Abstract

The search for genetic risk factors in type-II diabetes has been hindered by a failure to consider dietary variables. Dietary nutrients impact metabolic disease risk and severity and are essential to maintaining metabolic health. Genetic variation between individuals confers differences in metabolism, which directly impacts response to diet. Most studies attempting to identify genetic risk factors in disease fail to incorporate dietary components, and thus are ill-equipped to capture the breadth of the genome’s impact on metabolism. Understanding how genetic background interacts with nutrients holds the key to predicting and preventing metabolic diseases through the implementation of personalized nutrition. Dysregulation of iron homeostasis is associated with type-II diabetes, but the link between dietary iron and metabolic dysfunction is poorly defined. High iron burden in adipose tissue induces insulin resistance, but the mechanisms underlying adipose iron accumulation remain unknown. Hepcidin controls dietary iron absorption and distribution in metabolic tissues, but it is unknown whether genetic variation influencing hepcidin expression modifies susceptibility to dietary iron-induced insulin resistance. This review highlights discoveries concerning the axis of iron homeostasis and adipose function and suggests that genetic variation underlying dietary iron metabolism is an understudied component of metabolic disease.

## 1. Introduction

Diet influences metabolism, but individuals vary in metabolic response to diet, leading to different long-term health outcomes. The term gene-environment interaction (GEI) is applied to phenomena where two genotypes respond differently to identical environmental inputs, and to when identical genotypes respond differently to different environmental inputs. Put another way, genetic variation produces observable phenotypic differences under specific conditions, e.g., diet, temperature, or age [1].

While GEI connecting dietary iron and systemic metabolism are understudied, GEI connecting dietary iron and iron homeostasis have been observed. For example, a study examining the effectiveness of iron supplementation in a cohort of 334 anemic infants with low hemoglobin observed that, despite regular consumption of iron-fortified baby formula, only 34% of the infants showed improved iron status over a 6-month period [2]. Another study examined iron status in an elderly population (*n* = 1016), and found that 12.9% of individuals had clinically high levels of iron stores, despite normal levels of iron consumption [3]. These studies suggest there is substantial variation in iron homeostasis in human populations, and that GEI likely underlies individual response to dietary iron. Characterizing the genetic variation and dietary conditions that underlie differences in response to dietary iron may translate into therapeutic strategies for individuals with iron-associated metabolic disease. This review focuses on the role of iron in metabolic homeostasis and disease, and calls for researchers to incorporate dietary iron components when searching for genetic risk factors.

## 2. Diet and Diabetes: A Need for Dietary Perspective

Prevalence of diabetes mellitus type II (T2D) has risen since the 1960’s, while prevention and treatment remain an enormous challenge. There are a variety of “one-size-fits-all” treatments available, including exercise- and dietary-based interventions, prescription of Metformin, and weight loss surgery if T2D is accompanied by obesity. Unfortunately, even with strict adherence to these therapies, most patients are at risk of developing cardiovascular disease [4]. Appropriate biomarkers are needed to assess individual risk and prescribe appropriate precision therapeutic strategies. Genome-wide association studies (GWAS) have identified 8–30 loci associated with insulin resistance and T2D, but these loci only explain ~10% of the total heritability of the disease [5,6,7,8,9,10]. This is known as the problem of “missing heritability”, and can, in part, be explained by a failure to account for environmental influences.

Few studies have attempted to include environmental factors into GWAS analysis, uncovering GEI. Examples include polymorphisms in peroxisome proliferator-activated receptor gamma (PPARG) that increase fasting insulin levels only when high levels of polyunsaturated fats are consumed, and polymorphisms in zinc transporter SLC30A8, which are associated with elevated fasting glucose levels in a zinc-deficient diet [11,12]. Unfortunately, adding environmental variables to GWAS reduces the power to detect association and depends on participants to report dietary habits. Thus, candidate gene approaches have been adopted to examine the genetic underpinnings of T2D. Inbred mouse strains provide a valuable resource because of their high genetic and physiologic homology to humans and the ease of environmental manipulation. For example, a quantitative trait locus (QTL) associated with variation in triglycerides identified *HPX* (Hemopexin) as a top candidate gene in an F16 advanced intercross between the LG/J and SM/J strains of mice [13]. Hemopexin sequesters plasma heme, preventing oxidative stress and heme-bound iron loss. Functional follow-up of this candidate found that it impacts adipocyte differentiation, shows altered expression in obese adipose in both mice and humans, and is correlated with triglycerides [13]. Studies that leverage results generated by multiple experimental procedures, e.g., forward genetics screens, human and mouse gene expression analysis, and molecular functional studies, will shed light on the underlying mechanisms behind GWAS loci and highlight what genes may be critical under specific environmental conditions [14].

## 3. Dietary Iron: Too Much of A Good Thing?

Dietary iron is absorbed by duodenal enterocytes that express ferroportin, allowing iron to pass from the lumen of the gut into the bloodstream [15]. Plasma iron is bound by circulating transferrin, the primary iron transport molecule, and cells take in transferrin via transferrin receptors [16]. Upon entrance into the cell, iron is stored by the iron-chelating protein ferritin, which forms protein aggregates and prevents iron from engaging in redox reactions [17]. Iron-bound ferritin also circulates in the bloodstream, serving as a convenient metric for assessing total body iron stores. Iron storing tissues can release iron back into the bloodstream by expressing ferroportin [18].

Iron deficiency is the most common nutritional deficiency in the world, affecting about two billion people [19]. Iron deficiency is associated with metabolic complications including cardiovascular disease (CVD) and T2D, mainly through the development of anemia, where insufficient iron availability decreases red blood cell production [20,21]. Western countries have low levels of iron deficiency due to iron fortification of commonly consumed grains including wheat flour, corn meal, and rice [22]. Consumption of dietary iron has increased dramatically in the last 30 years, trending with the rise of T2D [23]. Patients with T2D have significantly elevated serum ferritin levels, and multiple studies associate dietary iron intake and diabetes risk [24,25,26,27]. Longitudinal analysis of iron-overloaded individuals found that patients in the highest quartile of serum ferritin levels have 3.5-fold greater risk of developing T2D over a 6-year follow-up period [28]. High serum ferritin is individually associated with increased BMI, hypertension, dyslipidemia, and reduced insulin sensitivity [29]. A limited number of studies have examined the reduction of serum ferritin by phlebotomy in patients with T2D with promising results, improving insulin sensitivity, insulin secretion and decreasing basal glucose levels [30]. Interestingly, ferritin is elevated during inflammation and infection, which complicates interpreting changes in ferritin levels in individuals with metabolic disease, who often have chronic inflammation [31]. Iron has been investigated as a risk factor in CVD, independent of T2D; however, results remain inconclusive [32]. Both iron deficiency and iron overload are implicated in metabolic disease, which underscores the importance of maintaining iron levels within a narrow physiological range. These studies connect iron stores and diabetes, but the genetic and physiologic mechanisms through which dietary iron contributes to T2D etiology are incompletely understood.

While all tissues require iron, the liver is the primary iron sensing organ. When the liver accumulates high levels of iron, the *HAMP* gene (hepcidin) is upregulated and secreted into the blood stream. Hepcidin binds and inhibits ferroportin in iron-storing tissues including the liver, adipose tissue, the spleen, macrophages, and duodenal enterocytes [33]. In enterocytes, inhibition of ferroportin by hepcidin reduces absorption of dietary iron into the blood stream by trapping it within the enterocyte, lowering circulating transferrin levels. Likewise, hepcidin inhibition of ferroportin in liver and adipose tissue prevents the export of iron in these cells, which increases iron retention within the tissue, and further decreases circulating transferrin availability. It follows that genetic variation in this pathway can lead to disruption of iron homeostasis.

Early evidence that dysregulation of iron homeostasis contributes to metabolic disease comes from the genetic iron-overload disorder hereditary hemochromatosis (HH). HH is characterized by loss of function mutations in the hepcidin pathway, leading to the inability to inhibit iron uptake and causing systemic iron overload. HH individuals have a 60% chance of developing T2D in their lifetime, compared to 35% in the general population [34]. Regular phlebotomy temporarily reduces circulating iron burden and blood glucose levels in HH subjects with T2D [35]. Mouse models of HH reveal that chronic iron overload decreases insulin secretion capacity due to high levels of oxidative stress [36]. While these studies connect iron stores and diabetes, the genetic and physiologic mechanisms through which dietary iron contributes to T2D etiology in non-HH individuals are incompletely understood.

A small number of studies have investigated the metabolic consequences of a high iron diet in mice, finding association with altered glucose and insulin homeostasis. C57BL/6J mice fed a high iron diet for 16 weeks had higher basal glucose levels, decreased insulin signaling assessed by phospho-Akt/Akt ratio, and impaired insulin tolerance assessed via an insulin tolerance test [37]. Another study found that C57BL/6J mice fed a combination high-iron/high-fat diet for seven weeks experienced greater dysregulation of insulin signaling than high-fat-fed mice alone, in part by increasing hepatic iron load, altering hepatic mitochondrial function, and increasing Interleukin-6 (IL6) inflammatory signaling. These results suggest dietary iron and fat work synergistically to alter systemic glucose metabolism [38]. Additionally, dietary iron restriction and iron chelation protected genetically obese *ob/ob* mice from insulin resistance and *β*-cell failure [39]. These studies indicate that iron overload can produce diabetic-like phenotypes by interfering with insulin sensitivity and hepatic gluconeogenesis, and by decreasing pancreatic *β*-cell insulin production. These studies are consistent with observations of dysregulated iron homeostasis in human subjects with T2D, and provide opportunities to study these mechanisms in depth. However, these studies do not incorporate multiple genetic backgrounds, so the breadth of the effect of dietary iron on metabolism may not be fully characterized.

## 4. Adipose Tissue: At the Intersection of Iron and Insulin Sensitivity

Adipose tissue is a dynamic endocrine organ that contributes to systemic glucose and insulin homeostasis by producing signaling molecules including adipokines in response to changes in energy and nutrient availability [40]. These adipose-specific functions influence systemic insulin sensitivity by altering the behavior of other metabolic tissues including skeletal muscle and liver. Increased iron burden in adipocytes negatively impacts adipokine signaling, implicating iron sequestration in adipose as a driver of insulin resistance [41,42].

Adipocytes produce the adipokine adiponectin in response to decreased energy availability, which promotes insulin sensitivity by stimulating translocation of glucose transporter GLUT4 to the cell membrane of skeletal muscle and liver [40]. Serum ferritin is negatively correlated with serum adiponectin in T2D patients [43], and phlebotomy increases adiponectin levels and improves insulin sensitivity, potentially through reduction of iron load. In vitro addition of physiologically high levels of iron to 3T3-L1 cells decreases adiponectin secretion through increased *Foxo1* expression, a transcription factor involved in adipocyte functions including adipogenesis, gluconeogenesis, and insulin signaling [34]. These studies reveal iron can directly impact adiponectin levels and alter systemic insulin sensitivity, and highlight key candidate genes that connect iron to adipose biology. Variants in these genes may contribute to variation in their function in response to iron stores. 

Leptin is another adipokine that directly influences insulin sensitivity by stimulating expression of *Glut4* in target tissues. Leptin also induces lipolysis and decreases lipogenesis in muscle and liver tissue which is critical to prevent lipotoxic accumulation of triglycerides, which is linked to insulin resistance [44]. C57BL/6J mice fed a high iron diet have decreased leptin expression in adipose tissue and lower circulating leptin levels. In vitro, treatment of 3T3-L1 adipocytes with ferric ammonium citrate (an iron source) decreased *Lep* expression by 48% and secreted leptin by 36% [45]. Further, leptin expression and protein level could be rescued by addition of deferoxamine, an iron chelator. Examination of the leptin promoter identified CREB binding, a responder to high cAMP levels, as an inhibitory mechanism in response to high iron conditions. In sum, high iron load in adipose tissue decreases leptin expression and protein levels, which in turn decreases systemic insulin sensitivity.

In contrast to adiponectin and leptin, the adipokine resistin is associated with decreased insulin sensitivity and negative health outcomes [46]. Resistin stimulates expression of suppressor of cytokine signaling 3 (*Socs3*) in mouse muscle tissue, which inhibits insulin receptor phosphorylation and activation of the insulin pathway. C57BL/6J mice fed a high iron diet show a 3.3-fold increase in serum resistin levels, and a subsequent 2.1-fold increase in adipose *Socs3* expression [37]. The mechanism through which iron induces resistin expression is not well characterized, but may reveal novel mechanisms in the relationship between iron and adipose health. Investigating how genetic variants in these key adipokines, adiponectin, leptin, and resistin, interact with dietary iron will open new avenues of research into adipose biology and iron’s effects on systemic metabolism. Further, investigating the cause of iron accumulation in adipose tissue promises to reveal novel risk factors associated with iron-induced insulin resistance.

## 5. Hepcidin: A Potential Link between Dietary Iron, Adipose Tissue, and Insulin Resistance

Hepcidin is a small peptide secreted by the liver that plays a regulatory role in iron metabolism. It binds and inhibits the iron exporter ferroportin, triggering endocytosis and degradation [33]. Loss of function mutations in the hepcidin pathway were independently identified as the cause of hemochromatosis (HH), a disease characterized by systemic iron-overload [47]. Proper hepcidin expression is integral to maintaining intracellular and plasma iron concentrations, making it an attractive candidate for investigating gene X dietary iron effects on metabolism.

The role of hepcidin in non-HH cases of T2D has been scantly investigated, with conflicting results. A cross-sectional analysis of 239 non-diabetic and 65 diabetic individuals revealed that diabetics had significantly lower circulating hepcidin levels, independent of inflammation status [48]. This study complements the observed high rate of diabetes in HH patients, who have reduced hepcidin expression. Other studies found that T2D patients have significantly higher serum hepcidin levels compared to healthy individuals, and serum hepcidin levels correlate with serum glucose levels [49,50]. However, a third study found no significant association between hepcidin and the onset of T2D [51]. While these findings seem contradictory, it may be explained by comparing systemic vs adipose-specific iron overload. Individuals with low hepcidin expression, including HH patients, are unable to inhibit enterocyte ferroportin, leading to continuous dietary iron absorption. This would result in systemic iron overload, where all tissues, including adipose, have a high iron burden. While adipose tissue can export iron via ferroportin, toxic accumulation of iron would occur due to the high concentration of circulating iron. Conversely, T2D individuals with high hepcidin expression have low ferroportin activity on enterocytes and adipose. In these patients, dietary iron absorption is low, but any circulating iron is sequestered in adipose tissue without means to be exported. This leads to iron-overload of adipose tissue with low iron availability for other tissues, causing the so-called “anemia of chronic disease”. Through systemic or adipose-specific mechanisms, both low and high levels of hepcidin expression can induce iron-overload in adipose tissue. These contradictory findings reflect the complex regulatory mechanisms that govern hepcidin expression.

**Figure 1:**
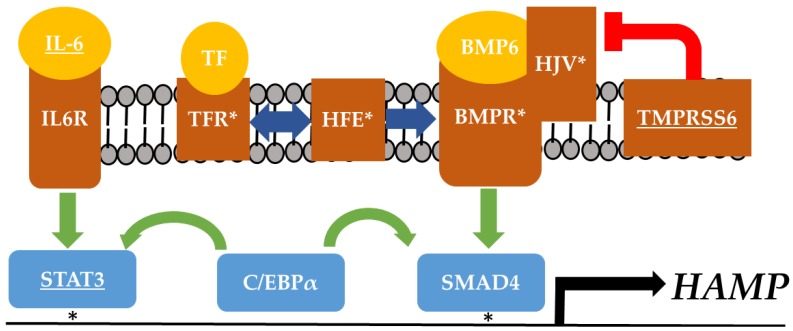
Hepatic hepcidin activation pathway. *HAMP* gene expression is activated by the IL6 and transferrin pathways, which are linked by C/EBPα. Genetic variants affecting protein function are associated with systemic iron overload (White Asterisks) and diabetes (Underlined), while variants in transcription factor binding sites in the *HAMP* promoter are associated with systemic iron overload (Black Asterisks). It is unknown if there are genetic variants in the *HAMP* pathway associated with diabetes risk during a high iron diet.

Hepcidin is regulated through two pathways, iron-sensing and inflammation-sensing, that converge on the 650 base pair proximal promoter region (Figure 1). The iron-sensing pathway is activated by circulating transferrin (TF), which binds to a transferrin receptor (TFR). This complex interacts with cofactors including HFE and hemojuvulin (HJV), which in turn sensitize BMP-receptor (BMPR) to BMP. Activation of BMPR activates the SMAD signaling pathway, resulting in SMAD4 binding at BMP response elements, increasing *HAMP* transcription [52]. Transmembrane Serine Protease 6 (TMPRSS6) inhibits HJV action, reducing BMP6R sensitivity. Loss of function mutations in HFE, HJV, TFR, and BMP receptor cause HH, while variants in TMPRSS6 are associated with reduced risk for T2D [53,54].

The inflammation-sensing pathway is less-well characterized. Interleukin-6 (IL-6) is upregulated in T2D, and is implicated in the development of insulin resistance. IL-6 binds to the IL-6 receptor, activating the JAK/STAT pathway, where STAT3 binds directly to the *HAMP* proximal promoter and increases hepcidin expression. C/EBPα is a highly expressed hepatocyte transcription factor, and is a necessary cofactor for IL-6 and transferrin stimulated activation of hepcidin [55]. Furthermore, hepcidin expression is directly regulated by insulin through STAT3 promoter binding and fasting insulin level is negatively correlated with *HAMP* mRNA expression in diabetic humans [56,57]. Taken together, the relationship between hepcidin and insulin sensitivity may be bidirectional, and is likely influenced by genetic variation in the hepcidin activation pathways.

Individuals vary greatly in total iron deposition and genetic variation in the hepcidin promoter is directly associated with iron status. A study examining the effects of promoter haplotypes on hepcidin gene expression in eight inbred strains of mice found several promoter polymorphisms in transcription factor binding sites that influence *Hamp* expression up to 3-fold [58]. In humans, three SNPs in the 5′ promoter region *of HAMP* are associated with severe iron overload including binding sites for SMAD and STAT3 (Figure 1) [59,60,61]. It is likely more regulatory variants influence hepcidin expression, and these may be context dependent. To date, no study has examined the role of hepcidin promoter polymorphisms in response to dietary iron. Polymorphisms in the hepcidin promoter can influence hepcidin expression and iron-loading in metabolic tissue, yet it remains unknown if hepcidin expression *per se* is a risk factor for T2D. Furthermore, it is unknown if, or to what degree, dietary iron facilitates this risk. Given the role of iron in the development of T2D, it is plausible that hepcidin expression may contribute to iron loading in adipose tissue leading to decreased insulin sensitivity and metabolic dysfunction. Identification and molecular characterization of genetic variants influencing hepcidin expression will provide new insights into the metabolic consequences of dietary iron in T2D.

## 6. Concluding Remarks

Prevention of diabetes remains a major challenge because of the polygenic nature of metabolic traits and the ability of diet to modify genetic risk. Dietary iron is an understudied component of this equation, despite the increase in iron fortification that correlates with increased T2D prevalence. Dysregulation of iron homeostasis is heavily implicated in metabolic disease, and high levels of dietary iron have been shown to induce insulin resistance through dysregulation of adipose tissue. Metabolic homeostasis is heavily influenced by genetic background, but the genetic architecture underlying iron’s influence on adipose tissue has not been investigated. Hepcidin is a promising candidate linking genetic variation, dietary iron, and adipose tissue function. These lines of evidence converge on a central question: how does genetic background modify the relationship between dietary iron and metabolic disease? Future efforts to uncover genetic risk factors for diabetes should consider including a dietary iron component. Dietary intervention studies in mice should include multiple genetic backgrounds to better characterize the range of dietary iron’s influence on metabolism. Combining these approaches will identify iron-related risk factors on a demographic level, and provide mechanistic insight into how genetic markers translate into metabolic response to diet.
